# Development of plant systemic resistance by beneficial rhizobacteria: Recognition, initiation, elicitation and regulation

**DOI:** 10.3389/fpls.2022.952397

**Published:** 2022-08-09

**Authors:** Lin Zhu, Jiameng Huang, Xiaoming Lu, Cheng Zhou

**Affiliations:** ^1^Key Lab of Bio-Organic Fertilizer Creation, Ministry of Agriculture and Rural Affairs, Anhui Science and Technology University, Bengbu, China; ^2^School of Life Sciences and Technology, Tongji University, Shanghai, China; ^3^Jiangsu Provincial Key Lab of Solid Organic Waste Utilization, Jiangsu Collaborative Innovation Center of Solid Organic Wastes, Educational Ministry Engineering Center of Resource-Saving Fertilizers, Nanjing Agricultural University, Nanjing, China

**Keywords:** induction of systemic resistance, microRNAs, beneficial rhizobacteria, syntaxins, volatile organic compounds

## Abstract

A plant growing in nature is not an individual, but it holds an intricate community of plants and microbes with relatively stable partnerships. The microbial community has recently been demonstrated to be closely linked with plants since their earliest evolution, to help early land plants adapt to environmental threats. Mounting evidence has indicated that plants can release diverse kinds of signal molecules to attract beneficial bacteria for mediating the activities of their genetics and biochemistry. Several rhizobacterial strains can promote plant growth and enhance the ability of plants to withstand pathogenic attacks causing various diseases and loss in crop productivity. Beneficial rhizobacteria are generally called as plant growth-promoting rhizobacteria (PGPR) that induce systemic resistance (ISR) against pathogen infection. These ISR-eliciting microbes can mediate the morphological, physiological and molecular responses of plants. In the last decade, the mechanisms of microbial signals, plant receptors, and hormone signaling pathways involved in the process of PGPR-induced ISR in plants have been well investigated. In this review, plant recognition, microbial elicitors, and the related pathways during plant-microbe interactions are discussed, with highlights on the roles of root hair-specific syntaxins and small RNAs in the regulation of the PGPR-induced ISR in plants.

## Introduction

Root exudates can shape highly specific micro-environments in plant rhizosphere, which are populated by a huge variety of soil-borne bacteria (Sasse et al., 2018; Pascale et al., 2020; Stassen et al., 2021). These root-associated bacteria are known as rhizobacteria, some of which can promote plant growth and are often referred to as plant growth-promoting rhizobacteria (PGPR) (Kloepper and Schroth, 1978). It has been well documented that PGPR can improve plant growth and stress adaptation by multiple strategies, such as secretion of hormone, reduction of host ethylene levels, and promotion of nitrogen fixation (Arshad et al., 2007; Reis and Teixeira, 2015; Backer et al., 2018). Besides their roles in plant growth promotion, PGPR can secrete antagonistic substances to suppress the growth of pathogens (Ajilogba and Babalola, 2013; Haskett et al., 2021).

Diverse biotic factors such as insects, bacterial and fungal pathogens, and viruses often lead to harmful effects, as reflected by biomass reduction, yield loss, and even plant mortality (Mertens et al., 2021). A vast number of PGPR strains such as *Bacillus*, *Pseudomonas, Enterobacter*, *Klebsiella*, *Azosprillum*, and *Paenibacillus* induce systemic resistance (ISR) of plants against biotic stress (Pérez-García et al., 2011; Beneduzi et al., 2012; Pieterse et al., 2014; Gkizi et al., 2016). PGPR can emit volatile organic compounds (VOCs) to stimulate the ISR responses in plants (Ryu et al., 2004). Different kinds of secondary metabolites such as bacterial quorum sensing (QS) molecules, siderophores and cyclic lipopeptides can also be released for provoking the ISR responses of plants by several defense-related signaling pathways (Aznar and Dellagi, 2015; Grady et al., 2016). Since the first report on the PGPR-induced ISR in plants (Van Peer et al., 1991), great progress has been made in understanding the mechanisms of recognition, initiation, elicitation, and regulation of plant ISR responses. In this review, we have provided an overview of the mechanisms and multiple processes associated with the PGPR-induced ISR in plants, and summarize recent advances about the roles of root hair-specific syntaxins and small RNAs in the process of the PGPR-mediated defense responses in plants.

## Recognition of plant growth-promoting rhizobacteria by host plants

Most of soil-borne microbes have no direct impacts on plant growth and fitness, but there are a large number of beneficial or pathogenic microbes among the huge diversity of plant microbiomes (Trivedi et al., 2020). Beneficial associations involve diverse microbes colonized in the rhizosphere, such as root-associated bacteria and fungi that promote plant growth (Lugtenberg and Kamilova, 2009; Niu et al., 2020). Since beneficial microbes are considered as alien organisms, the active interference to plant defense systems is the basis for establishing a close and mutually beneficial relationship with the hosts. Like animals, plants have an innate immune system that is activated after identifying invading organisms. Recognition of non-self-signal molecules is a key step to achieve effective defense responses, which can be recognized by pattern recognition receptors (PRRs) in plants. Microbial associated molecular patterns (MAMPs), commonly called as pathogen associated molecular patterns (PAMPs), can be perceived by these PRRs (Dodds and Rathjen, 2010; Sanabria et al., 2010). PRRs recognize the MAMPs/PAMPs and further activate the PAMP-triggered immunity (PTI), conferring the first line of plant defense against pathogens. In plants, the most characteristic PRRs are the receptor like proteins (RLPs) or the receptor-like kinases (RLKs) (He and Wu, 2016; Tang et al., 2017). RLKs are the putative transmembrane proteins, harboring both extracellular ligand recognition and intracellular kinase domains that are responsible for signal transduction. The structures of RLPs are much similar, but lack kinase domains (Walker, 1994).

Plants can recognize common structures from different microbial species. Many common MAMPs recognized by plants have been documented, such as chitin, lipopolysaccharides (LPS), flagellin, and peptidoglycan (Mishra et al., 2012; Newman et al., 2013; D’Ambrosio et al., 2017; Ma et al., 2021). In nature, plants can not only interact with pathogens, but also form beneficial interactions with soil-borne microbes. Typical examples of symbiotic plant-microbe combinations are mycorrhizal fungi that form symbiotic relationships with many plant species and help absorb water and minerals, rhizobia that fix atmospheric nitrogen for plants, and PGPR that improve plant growth and inhibit disease occurrence (Lugtenberg and Kamilova, 2009; Suzaki et al., 2015; Lindström and Mousavi, 2020). Many of them exist outside the plant roots, while others are endophytic microbes that establish a closer relationship with the hosts. Due to many MAMPs that are widely existed and preserved in microbes, beneficial microbes display the similarity with pathogens (Hacquard et al., 2017). In order to achieve benefit services from these beneficial microbes, it is important for plants to identify the differences between pathogenic and beneficial microbes. Accumulative evidence has indicated that beneficial microbes were initially regarded as potential invaders, leading to activation of plant immunity (Pieterse et al., 2012). However, like pathogens, many PGPR strains can inhibit host defensive responses and thus establish successful relationships with their hosts. In addition, beneficial microbes seem to have similar strategies to avoid plant recognition systems (Park and Ryu, 2021).

PGPR are beneficial microbes, which establish symbiotic or non-symbiotic associations with their hosts and improve plant growth. PGPR can produce massive MAMPs (e.g., flagellin and LPS) to stimulate plant defense (Zamioudis and Pieterse, 2012). Different PGPR strains can be recognized by plant defense systems and trigger defense responses in the early stage in a way similar to PTI (Jacobs et al., 2011). LPS derived from cell wall of *Pseudomonas fluorescens* WCS417 is composed of lipid A/innercore/O-antigen side chains, which enhances plant defense against *Fusarium* pathogens (Leeman et al., 1995). However, unlike the PTI triggered by pathogens that usually lead to severe cell damage, the PGPR-mediated defense responses are transient and mild for establishing reciprocal relationships with the hosts. The flg22 peptide from beneficial *Burkholderia* species slightly induces oxidative burst and transiently activates the expression of defensive genes without repression of plant growth (Felix et al., 1999). *Pseudomonas fluorescens* WCS417r can inhibit the flagellin-triggered PTI reaction in *Arabidopsis* roots by secreting small molecular compounds (Millet et al., 2010). The colonization of PGPR on the roots requires local inhibition of PTI to protect PGPR from antibacterial compounds triggered by MAMPs, indicating that a coevolution results in the regulation of plant defense after perceiving specific signals of beneficial microbes.

## Initiation of plant induce systemic resistance by plant growth-promoting rhizobacteria

In plants, PRRs can recognize common microbial signals, such as PAMPs and MAMPs (Lu and Tsuda, 2021). To achieve successful invasion, pathogens have evolved to weaken the activation of host immune systems. Moreover, it can deploy virulence effector proteins to inhibit the PTI signaling pathway or avoid recognition by the hosts (Zipfel, 2009; Hatsugai et al., 2017; Kud et al., 2019). Subsequently, a second line of defense has been acquired, in which specific effector molecules from pathogens are perceived by the nucleotide-binding leucine-rich repeat (NB-LRR) receptor proteins, leading to effector-triggered immunity (ETI). The gene-for-gene resistance in plants belongs to the ETI, usually accompanied by programmed cell death at infecting sites, thereby preventing the entry of biotrophic pathogens (Dodds and Rathjen, 2010). The occurrence of PTI and ETI often stimulates the ISR responses in plant tissues far from the pathogen-infecting sites and involves distant signals that propagate the enhanced defense in intact parts of plants. The classical mode of pathogen-induced resistance is often called as systemic acquired resistance (SAR), which confers the increased resistance of plants against diverse pathogens (Vlot et al., 2009). Like the pathogen recognition systems, herbivorous insects can be recognized by host plants, probably by similar signaling pathways (Pan et al., 2016; Aljbory and Chen, 2018).

Due to its broad-spectrum effectiveness, the pathogen-induced SAR is initially thought to be similar to the PGPR-induced ISR in the mechanistic way. Root colonization by PGPR induces a state of priming in host plants, in which plants can respond stronger and faster to pathogenic attacks, reflecting a common feature of ISR triggered by PGPR (Pieterse et al., 2014; Gkizi et al., 2016; Fan et al., 2018; Zehra et al., 2021). To date, diverse PGPR strains have been shown to provoke ISR in plants, which confers broad-spectrum disease resistance (Nishad et al., 2020; Samaras et al., 2021). Several PGPR strains can stimulate the SA-dependent ISR responses, which are similar to the SAR. *Pseudomonas aeruginosa* 7NSK2 that is not able to produce SA can increase the resistance of bean plants against pathogens, but not observed for the *NahG*-overexpressing plants (De Meyer et al., 1999; Audenaert et al., 2002). Also, *P. fluorescens* strain P3 overexpressing the biosynthetic gene of SA can trigger the SAR in plants (Maurhofer et al., 1998). In the cases that PGPR induces the SAR, the accumulation of reactive oxygen species (ROS) is essential for activating the SA-dependent SAR in plants (De Meyer et al., 1999; Niu et al., 2016a; Zehra et al., 2021). *Bacillus cereus* AR156 can stimulate the SAR responses by activating the SA signaling pathway in an NPR1-dependent manner (Niu et al., 2016a). *Pseudomonas* sp. 23S induces ISR in tomato plants, which is closely related to upregulation of *PR1a* transcripts (Takishita et al., 2018). More recently, several *Bacillus* strains have been reported to induce host ISR against the pepper bacterial spot disease by increasing the expression of *PR* genes such as *CaPR1*, *CaPR4*, and *CaPR10* (Li et al., 2020). However, activation of the SA-independent ISR responses by PGPR also occurs in different plant species. *P. fluorescens* WCS417r triggers the ISR of radish plants against *Fusarium oxysporum* without increasing the expression of *PR* genes, which is a typical characteristic of SAR. Similarly, the WCS417r-induced ISR in *Arabidopsis* plants is not associated with the up-regulation of *PR* genes in leaves (Pieterse et al., 1996). The assays of *NahG*-overexpressing plants that are deficient in the accumulation of SA reveal that the WCS417r-induced ISR responses are independent on the SA signaling pathway (Pieterse et al., 1996, 2000). Similar phenomenon is also observed for *Pseudomonas putida* WCS358r-mediated activation of ISR responses in plants (Kloppholz et al., 2011). Besides the plant hormone SA, jasmonic acid (JA) and ethylene (ET) are essential for regulating plant defense responses. In *Arabidopsis* mutants deficient in the JA/ET signaling, the PGPR-induced ISR in plants is largely compromised (Pieterse et al., 1998). The WCS417r-induced ISR responses are defective in both the JA (e.g., *coi1*, *jin1*, and *jar1*) and ET signaling mutants (e.g., *eir1*, *etr1*, and *ein3*) (Pieterse et al., 1998; Knoester et al., 1999; Pozo et al., 2008). Mounting evidence has indicated that JA and ET are essential for activating the SA-independent defense responses induced by beneficial microbes. The PGPR-induced ISR responses are effective against pathogens and insect herbivores that are sensitive to the JA/ET-dependent defense (Van Wees et al., 2008; Pineda et al., 2010). Interestingly, the SA- and JA/ET-dependent signaling pathways are also involved in the regulation of the PGPR-induced ISR in plants. Both the SA and ET signaling are involved in the regulation of *Bacillus velezensis* CLA178-mediated ISR in *Rosa multiflora* (Chen et al., 2020). *B. amyloliquefaciens* CRN9 can trigger innate immunity and inhibit virus growth in plants *via* ISR activated by both the SA and JA/ET signaling pathways (Rajamanickam and Nakkeeran, 2020). Therefore, different PGPR strains can trigger host ISR against the attacks of pathogens and insects, involving activation of intricate signaling networks.

**FIGURE 1:**
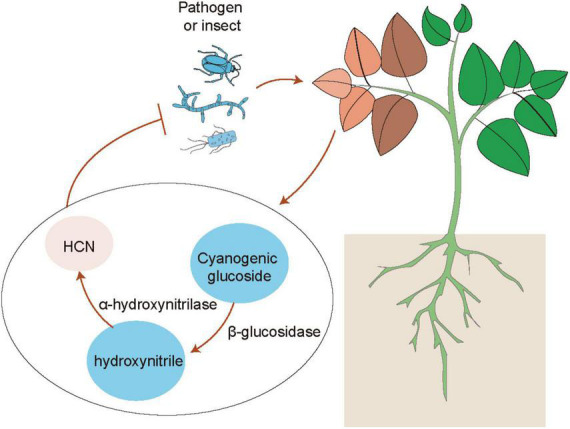
Mechanisms of hydrogen cyanide (HCN)-mediated plant defense. When plants are subjected to the attacks of herbivores and pathogens, the accumulation of cyanogenic glucosides in leaves is quickly catalyzed by two key enzymes including β-glucosidases and α-hydroxynitrilase into producing toxic HCN, which confers the enhanced plant defense against herbivores and pathogens.

**FIGURE 2:**
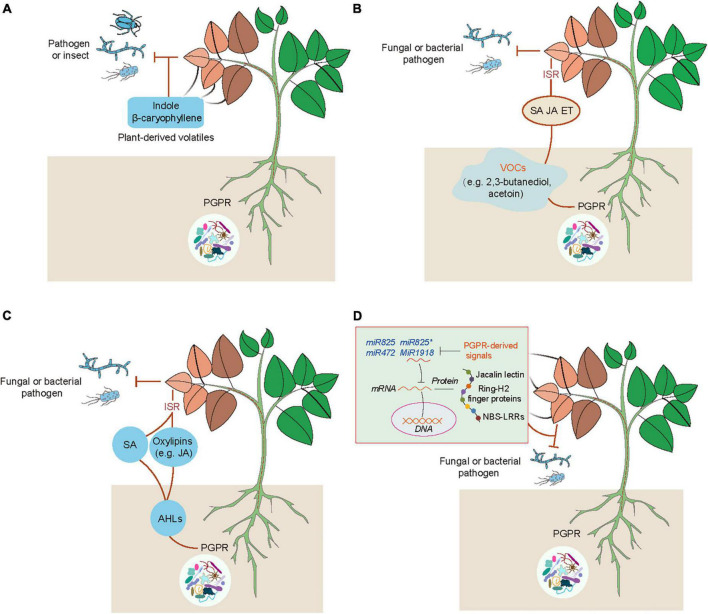
Multiple acting models of PGPR-induced ISR in plants. **(A)** PGPR-induced releases of plant volatiles act as defense-related substances against biotic stress. PGPR can induce the emission of plant volatiles such as indole and β-caryophyllene that enhance plant defense against the attacks of pathogens and insect pests. **(B)** PGPR can emit various kinds of VOCs such as 2,3-butanediol and acetoin, which lead to activation of hormone (SA, ET, and/or JA) signaling pathways that provoke plant ISR against pathogens. **(C)** PGPR can secrete the QS molecules (N-acyl-homoserine lactones, AHLs) to activate the SA- and JA-mediated pathways, MAPK cascades, and oxylipin-induced defense responses (e.g., promotion of stomatal closure, and the increased accumulation of callose, lignin, ROS and phenolic compounds). These effects lead to the enhanced plant defense against pathogens. **(D)** Suppression of plant miRNAs by PGPR enhances plant defense against pathogenic attacks. PGPR can release certain signals to repress negative regulators of plant defense systems such as miRNA825/miRNA825*, miR472 and miR1918, and thus enhance the expression of defense-related genes associated with jacalin lectin, Ring-H2 finger gene, and NBS-LRRs, which contribute to the increased resistance of plants against pathogens.

Emerging evidence has indicated that plants can use volatiles as the language to communicate with diverse microbes (e.g., bacteria, fungi and virus), insects and other neighboring plants (Ballhorn et al., 2013; Simpraga et al., 2016; Sharifi et al., 2018; de Almeida et al., 2021). Several kinds of volatiles (e.g., α-pinene and isothiocyanate) are often liberated by plants, which function as the cues for attracting insects (Elzen et al., 1983). Injured plants release different kinds of volatiles such as C6 fatty acid derivatives, isoprenoids (terpenes), methyl salicylate and indoles, which can be perceived by neighboring plants (Simpraga et al., 2016). Volatile cyanogen such as hydrogen cyanide (HCN) is also an important defense metabolite in plants (Figure 1). The production of cyanogenic glucosides by many plant species can be catalyzed by two key enzymes including β-glucosidases and α-hydroxynitrilase to release toxic HCN in response to insect herbivory and pathogens (Clausen et al., 2015; Hansen et al., 2018; Olsen et al., 2021). Although the release of HCN occurs only in response to cell injury, cyanogenesis has been considered as a constitutive plant defense (Pentzold et al., 2014; Bjarnholt et al., 2018). In addition, HCN can also act as a signaling molecule in plants, similar to other molecules such as nitric oxide (NO) and hydrogen sulfide (H_2_S) (Aroca et al., 2018; Feng et al., 2019; Wei et al., 2020). These signaling molecules can induce post-translationally modification of proteins including nitrosylation and persulfidation (Aroca et al., 2018; Gupta et al., 2020). HCN can promote the S-cyanylation of proteins by adding the SCN groups to cysteines, which leads to the alteration of protein functions (Gotor et al., 2019). Moreover, JA is also involved in the regulation of plant cyanogenesis and thus increases the resistance of lima bean to insect herbivory (Kautz et al., 2014). Interestingly, plants can emit similar kinds of volatiles after pathogenic attacks or inoculation with PGPR (Ballhorn et al., 2013). PGPR can regulate plant physiological processes and stress adaptation through different ways such as promotion of nutrient uptake and mediation of hormone signaling pathways (Sharifi and Ryu, 2017). Several PGPR strains have also been demonstrated to enhance plant defense against pathogens and insect pests by modification of host volatile profiles. Co-treatment with rhizobia and JA markedly reduces the emission of volatiles, but promotes the release of shikimic acid derivative indoles (Figure 2A). Indoles have been reported to mediate plant growth, disease resistance and bacterial pathogenesis (Lee et al., 2015; Sharifi and Ryu, 2017). It can also provoke the ISR responses of plants against herbivores by promoting the biosynthesis of terpenes and JA isoleucines in leaves. Veyrat et al. (2016) have shown that the indole-deficient plants exhibit the increased attractiveness to *Spodoptera littoralis* caterpillars. Before the attacks of *Mamestra brassicae*, pretreatment of *Arabidopsis* plants with *Pseudomonas simiae* WCS417r activates the ORA59-mediated JA/ET signaling pathways. Plants inoculated with WCS417r exhibit the increased attractivity to the parasitoid *Microplitis* mediator with less lilial, (E)-a-bergamotene and MeSA emissions compared with the control plants (Pangesti et al., 2015). Transcriptional changes of several genes associated with the biosynthesis of oxylipins are considerably induced by *P. putida* KT2440, which are closely related to the enhanced plant defense against pathogens and insects. The release of two defense-related volatiles including β-caryophyllene and indoles is remarkably promoted by the strain KT2440 (Planchamp et al., 2014).

## Microbial elicitation of plant induce systemic resistance

A large number of MAMPs from beneficial microbes such as flagellin, liposaccharides (LPS), peptidoglycans, and hairpins induce the MAMP-triggered plant immunity to inhibit pathogen infection (Newman et al., 2013; D’Ambrosio et al., 2017; Ma et al., 2021). The cascade of events closely related to plant ISR and SAR is activated by some regulatory genes such as MAPKs, WRKYs and MYCs (Kazan and Manners, 2013; Meng and Zhang, 2013; Jiang et al., 2016). In *Arabidopsis*, *P. putida* WCS358-derived flagellin can trigger ISR against *Pseudomonas syringae*. Similarly, LPS, tripartite amphipathic molecules with O-antigen side chains, from several PGPR strains can also enhance plant defense against pathogenic attacks (Meziane et al., 2005). Furthermore, diverse kinds of active molecules such as VOCs, QS molecules, siderophores, and cyclic lipopeptides can be released by PGPR and function as important ISR elicitors.

### Bacterial volatile organic compounds

Plants are inevitably subjected to VOCs from diverse organisms including bacteria, fungi and neighboring plants (Bailly et al., 2014; Karamanoli et al., 2020; Vlot et al., 2021). More than 1000 VOCs (such as alcohols, alkenes and ketones) and non-organic compounds (such as HCN and NH_3_) can be liberated by a huge number of microbes (Audrain et al., 2015). Bacterial VOCs function as important regulators for plant growth and stress resistance (Sharifi and Ryu, 2016). Different PGPR strains can emit differential blends of VOCs, which are involved in the regulation of bacterial life cycles and interactions with their hosts. VOCs have also been reported to regulate the antibiotic sensitivity, motility and biofilm formation of bacteria, which function as the virulence-mediating factors for bacterial pathogens (Sharifi and Ryu, 2016). Indeed, single volatile compound can also benefit the emitters (Huang et al., 2012; Bailly et al., 2014). Bacteria-released indoles can promote the formation of bacterial biofilm, antibiotic resistance, and kill nematodes (Anyanful et al., 2005; Bailly et al., 2014; Audrain et al., 2015).

Bacterial VOCs are key inducers for stimulating the ISR responses of plants against pathogen infection (Figure 2B). Different PGPR strains can liberate diverse kinds of VOCs, which induce systemic defense of plants against pathogenic attacks in a strain-specific manner (Ryu et al., 2004; Song and Ryu, 2013; Sharifi and Ryu, 2016; Vlot et al., 2021). *Bacillus subtilis* GB03-emitted VOCs trigger the ISR responses of *Arabidopsis* plants to inhibit the attacks of *Erwinia carotovora* subsp. carotovora by activation of the ET signaling pathway rather than the JA and SA signaling pathways (Ryu et al., 2004). The release of VOCs by *B. subtilis* FB17 induces the ISR responses of *Arabidopsis* plants against the hemibiotrophic pathogen *P. syringae* pv. tomato (*Pst*) DC3000, which is attributable to the stimulation of both the SA and ET signaling pathways (Rudrappa et al., 2010). Bacterial VOCs can enhance the expression of *PDF1.2* and *PR1*, and provoke both the SA- and JA-dependent pathways, which contribute to the increased host defense against pathogenic attacks (Sharifi and Ryu, 2016). It is increasingly evidenced that bacterial VOCs regulate multiple signaling pathways for enhancing plant defense. The release of 2,3-butanediol and acetoin by PGPR increases the resistance of plants against pathogen infection (Farag et al., 2006). Treatment with acetion greatly induces the expression of *PR-4* and SA-related signaling pathways in *Agrostis stolonifera* (Cortes-Barco et al., 2010a). However, 2,3-butanediol induces systemic resistance of *Nicotiana benthamiana* against the fungal pathogen *Colletotrichum orbiculare* by activating the JA-dependent pathway, but not the SA-dependent pathway (Cortes-Barco et al., 2010b).

Recent genome sequencing have revealed that many bacterial species such as *Deinococcus radiodurans*, *Bacillus halodurans*, and *B. subtilis* possess the NOS-like proteins, which are essential for generating the gaseous molecule NO (Adak et al., 2002). NO is one of the most important bacterial VOCs that induce the plant ISR against microbial pathogens (Trapet et al., 2015). The mechanisms by which NO regulates plant defense signaling cascades have been well examined. The S-nitrosylation of proteins is an important regulatory event modulated by NO, in which NO can react with the cysteine-rich thiol groups in proteins to form the S-nitrosothiols (Yu et al., 2014). It has been well documented that several transcription factors can be S-nitrosylated in plants (Castillo et al., 2015; Kawabe et al., 2018). In *Arabidopsis*, NO can switch the translocation of the SA signaling component, NPR1, which takes part in the induction of *PR* genes, into the nucleus (Tada et al., 2008; Lindermayr et al., 2010). The S-nitrosylation of the zinc finger transcription factor SRG1 plays a critical role in regulating plant defense responses (Cui et al., 2018). Furthermore, the modified activity of the *Arabidopsis* NADPH oxidase, AtrbohD, is a typical example of the role of S-nitrosylation in plant defense (Yu et al., 2014; Sivakumaran et al., 2016). It has been clearly evidenced that SAR can be initiated by NO, which works together with ROS and SA signals (Wendehenne et al., 2014). In addition, NO has also been demonstrated to interact with both the JA and ET signaling pathways for regulating plant defense responses (Mur et al., 2013).

### Bacterial quorum sensing molecules

QS is a wide-existed biological process, in which bacteria can synthesize and perceive QS molecules to mediate their cell density and collective behaviors (Papenfort and Bassler, 2016; Abisado et al., 2018; Mukherjee and Bassler, 2019). The secretion of N-acyl-homoserine lactones (AHLs) by Gram-negative bacteria can function as QS molecules to regulate the intra-population communications (Fu and Dong, 2013; Ortiz-Castro and López-Bucio, 2019). Bacteria can perceive the QS molecules for activating or inactivating the expression of several genes related to diverse processes such as biofilm formation and chemotaxis (Bellezza et al., 2014; Laganenka et al., 2016; Zhang et al., 2020). AHLs are one of the well-examined QS molecules that harbor an acyl side-chained homoserine lactone ring. The hydrogen at the C3 position from different length of the acyl chains can be substituted with a hydroxyl or a ketone group. The lactone ring is essential for the recognition of AHLs by its cognate receptors, and the specificity of cell-to-cell recognition and interaction is determined by both the fatty acid chain length and amide group (Whitehead et al., 2001; Churchill and Chen, 2011).

Bacterial QS molecules are also involved in the mediation of plant behaviors (Ortiz-Castro and López-Bucio, 2019). Although the mechanisms underlying plants perceive the QS molecules remain largely unclear, AHLs can regulate gene expression, protein profiles and root growth (Ortiz-Castro et al., 2008; Schenk et al., 2012). Proteomic analysis of the roots of *Medicago truncatula* has revealed that treatment with different AHLs changes the expression of 150 proteins involved in multiple processes such as flavonoid synthesis and oxidative stress (Mathesius et al., 2003). Similarly, treatment with oxo-C8-HSL remarkably induces the expression of proteins associated with carbon metabolism and plant defense in *Arabidopsis* seedlings (Ding et al., 2016). Shrestha et al. (2020) have shown that different forms of AHLs can trigger specific responses in plants, which depend on the length of the acyl moiety. Primary root growth can be promoted by the short acyl-chained AHLs, which is mainly attributable to activation of auxin signaling pathways (Von Rad et al., 2008; Schenk et al., 2012). The expression of several genes related to hormone signaling pathways is markedly increased in plants treated with the N-3-oxohexanoyl homoserine lactone (oxo-C6-HSL) (Von Rad et al., 2008). The increased auxin level is essential for promoting the formation of adventitious roots in *Vigna radiate* treated with the N-3-oxo-decanoyl-homoserine-lactone (3-O-C10-HL) (Bai et al., 2012).

Besides the roles of AHLs in the regulation of plant growth, AHLs can effectively ISR in plants (Figure 2C). The long acyl-chained AHLs have been reported to stimulate the ISR in different plant species (Schikora et al., 2011; Shrestha et al., 2019). The enhanced defense of AHL-treated plants is closely related to activation of multiple signal components. N-hexanoyl-homoserine lactone (HHL) promotes the biosynthesis of SA in plants, which contributes to enhancing the resistance of plants against *Alternaria alternate* (Schuhegger et al., 2006). N-decanoyl-homoserine lactone increases the resistance of tomato plants against *Botrytis cinerea* by activation of the JA signaling pathway (Hu et al., 2018). Exposure to N-3-oxo-tetradecanoyl-homoserine lactone (OTHL) provokes the mitogen-activated protein kinase (MAPK) cascades and thus enhances the transcription of defense-related transcription factors, thereby increasing the resistance of *Arabidopsis* and barley plants against obligate biotrophic fungi (Schikora et al., 2011). In addition, oxo-C14-HSL stimulates the production of oxylipins and further promotes the accumulation of callose and phenolic compounds, and stomatal closure, which result in the increased resistance of *Arabidopsis* plants against pathogen infection (Schenk and Schikora, 2014). The oxo-C14-HSL-induced disease resistance of cucumber plants has also been found to be associated with the enhanced deposition of lignin and callose, phenolics and ROS levels, and defense-related enzymatic activities (Pazarlar et al., 2020).

### Siderophores

Iron (Fe) is an indispensable element for all living creature because of its redox catalyzing ability, while excess Fe often triggers overproduction of hydroxyl radicals that are harmful to cell metabolism and structures (Verbon et al., 2017). Dynamic regulation of Fe homeostasis is the most critical mechanism for mediating plant-pathogen interactions (López-Berges et al., 2013; Aznar et al., 2015; Liu et al., 2021). Plants can employ a Fe-withholding strategy to weaken pathogen virulence or elevate Fe levels for inducing oxidative burst (Cassat and Skaar, 2013). Many studies have confirmed that siderophores are secreted by plant pathogens to fight for Fe with host plants and attenuate the Fe-regulated immune responses. Beneficial soil bacteria can suppress plant disease occurrence by reducing the bioavailability of Fe in the rhizosphere (Loper and Buyer, 1991; Berendsen et al., 2015). They can also directly stimulate the ISR in plants by activating the Fe uptake-associated signaling pathways (Oide et al., 2006; Lemanceau et al., 2009). In the mammalian immune systems, the Fe-withholding strategy facilitates the hosts to prevent pathogen infection (Meziane et al., 2005; López-Berges et al., 2012). By contrast, the functions of Fe in plant defense responses are even more intricate, since it involves a tripartite interaction in the rhizosphere among beneficial bacteria, pathogens and plants (Harman et al., 2004; De Vleesschauwer et al., 2008; Verbon et al., 2017).

Plant pathogens can secrete siderophores to acquire Fe, which is required for their virulence and successful invasion (Martinez-Medina et al., 2016; Gu et al., 2020). Before pathogen infection, soil-borne pathogens need to fight for the scarcely available Fe in the rhizosphere with other microbes for growth. Microbial release of siderophores has been demonstrated to play vital roles during the warfare for rhizopsheric Fe (Expert et al., 1996; Lamont et al., 2002; Weller et al., 2002; Pozo et al., 2008; Taguchi et al., 2010; Franza and Expert, 2013; Aznar et al., 2015). The efficiency of siderophore-mediated acquisition for Fe is mainly attributable to the affinity of siderophores for Fe, their species-specificity and abundance. The siderophore-recognized receptors of microbes are highly specific, while different microbes can also recognize and assimilate heterologous siderophores (Loper and Buyer, 1991). Highly rhizosphere-competent microbes can synthesize and release specific siderophores and possess various receptors for recognizing heterologous siderophores (Berendsen et al., 2015). PGPR can secrete such high-affinity Fe uptake and species-specific siderophores to compete for Fe with soil-borne pathogens, thereby reducing plant disease occurrence (Lemanceau et al., 2009). During long-term evolution, soil-borne pathogens also develop adaptive strategies to confront antagonistic microbes. In plants, cellular Fe homeostasis can be mediated by plant pathogens and rhizosphere microbes (Oide et al., 2006; López-Berges et al., 2012). Many studies have unraveled a close connection between the Fe homeostasis and PGPR-induced ISR in plants. The release of siderophores by PGPR effectively elicits ISR in plants (Meziane et al., 2005; De Vleesschauwer et al., 2008; Grady et al., 2016; Fan et al., 2018; Li et al., 2020). Other beneficial fungi such as *Piriformospora indica* and *Trichoderma* species can also mediate the uptake of Fe and trigger the ISR in host plants (Harman et al., 2004; Peskan-Berghofer et al., 2004). However, the underlying mechanisms behind the associations between cellular Fe status and PGPR-induced ISR in plants remain obscure.

**FIGURE 3:**
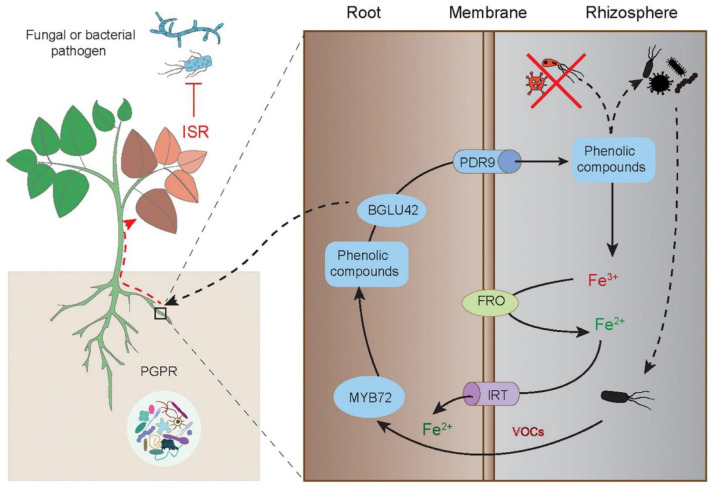
Elicitation of the MYB72-mediated ISR in plants by PGPR-released VOCs. The root-specific gene MYB72 initiates the ISR and Fe uptake in plants induced by PGPR. The ISR-eliciting PGPR can activate the expression of MYB72, which controls the biosynthesis of fluorescent phenolic compounds, and the expression of BGLU42 encoding the glucose hydrolase gene and PDR9 encoding the ABC transporter gene, thereby triggering root exudation of phenolics. The root-released phenolics further promote the mobilization of Fe^3+^ and make it available for reduction and uptake by plant roots. The phenolics can also shape specific rhizosphere microbiota. Moreover, the MYB72-dependent BGLU42 activity is essential for stimulating the ISR responses. This figure is acknowledged by Verbon et al. (2017).

The molecular basis of initiation, signaling transduction and activation of ISR in plants has been well illustrated in the interactions between *Arabidopsis* plants and *P. fluorescens* WCS417 (Figure 3). In the plant-microbe system, the disease resistance of ISR-expressing plants is closely related to activation of both the JA and ET signaling pathways. However, the PGPR-induced ISR responses are not attributable to the promoted biosynthesis of these hormones or substantial expression of defense-related genes in plants. Conversely, a quicker and stronger stimulation of defense responses is observed in the ISR-expressing plants upon exposure to insect or pathogenic attacks (Pieterse et al., 2014). Although no marked alterations of transcriptome changes occur in the WCS417-colonized *Arabidopsis* leaves, bacterial colonization leads to massive changes in the roots (Verhagen et al., 2004). MYB72, plays a regulatory role in the metabolism of Fe-mobilizing phenolics under Fe deficiency, is among differentially expressed genes in the WCS417-treated roots (Zamioudis et al., 2015). Other beneficial microbes such as *Trichoderma* species also result in similarly changing patterns of *MYB72* expression, but that is not found in the plants colonized by the non-ISR-inducing strain *P. fluorescens* WCS374 (Van der Ent et al., 2008; Berendsen et al., 2015). Treatment with WCS417 or *Trichoderma* species is not able to induce the establishment of ISR in the roots of the *Arabidopsis myb72* mutant, indicating that MYB72 is essential for initiating ISR provoked by beneficial microbes (Segarra et al., 2009). MYB72 can directly regulate the expression of *BGLU42* and *PDR9* genes, which are required for root exudation of Fe-mobilizing phenolics under Fe deficiency (Zamioudis et al., 2014). Interestingly, phenolic compounds have been shown to be considerably secreted by the roots inoculated with PGPR (Van de Mortel et al., 2012; Zhou et al., 2016). The *Arabidopsis bglu42* mutant cannot initiate the ISR responses upon exposure to WCS417, and overexpression of *BGLU42* in *Arabidopsis* increases the resistance against broad-spectrum pathogens (Zamioudis et al., 2014). Therefore, the PGPR-induced ISR responses are closely related to the MYB72-regulated phenolic metabolisms and Fe uptake.

### Cylic lipopeptides

Cylic lipopeptides (CLPs) such as iturin, surfactin, and fengycin exhibit antibacterial activities, which can be generated by different *Bacillus* species (Raaijmakers et al., 2010). The surfactin family contains heptapeptide, which is linked to β -hydroxyl fatty acid chains with a length of 12–16 carbon atoms to form a ring lactone ring structure, and shows strong antimicrobial activity, but no obvious antifungal activity (Henry et al., 2011). Surfactin and fengycin (but not Iturin) trigger the plant ISR against fungal pathogens, although they show differential preferences for different plant cell types (Ongena and Jacques, 2008).

During extracellular matrix formation, the surface proteins secreted by *B. subtilis* can also function as signaling molecules to mediate ISR, root colonization, and biofilm formation (Shank and Kolter, 2011; Arnaouteli et al., 2021). *B. amyloliquefaciens* FZB42 is a natural isolate, which can stimulate plant growth and produce three families of lipopeptides including surface proteins (the surfactin family), bacilomycin D (the iturin family), and fonamycin (the fengycin family) (Koumoutsi et al., 2004; Idris et al., 2007). The secretion of surface proteins from *B. amyloliquefaciens* into perennial ryegrass is important for activating the plant ISR against rice blast infection. Surface protein molecules are mainly bound to cell membranes, and the perception of surface proteins by plant cells contributes to their ISR activity (Henry et al., 2011). This perception is required for sensitizing the activity of resistance in plants, which leads to the surveillance state being extremely sensitive to the penetration of fungal pathogens, thereby provoking rigorous stimulation of H_2_O_2_-mediated plant defense. However, the fungal pathogen *Magnaporthe grisea* induces changes in the metabolic profiles of host cells (Parker et al., 2009), and *M. grisea* can manipulate antioxidant systems to weaken the H_2_O_2_-mediated defense in plants (Chi et al., 2009). Thus, the activated H_2_O_2_-mediated defense in plants is essential for restricting pathogen proliferation. Samalova et al. (2014) have reported that the *M. oryzae* redox potential exceeds the H_2_O_2_-mediated oxidation potential in non-induced susceptible plants (Samalova et al., 2014). The surfactin-treated perennial ryegrass exhibits a rapid and powerful induction of H_2_O_2_-mediated defense responses, which contributes to the enhanced resistance against gray leaf spot disease.

In plants, oxylipins are a series of lipid metabolites generated from the oxidation of polyunsaturated fatty acids, which function as antimicrobial substances and signaling molecules that induce defense responses and regulate cell death (Griffiths, 2015). Interference with the oxylipin pathways such as the biosynthesis or perception of oxylipins affects plant defense against pathogens (Prost et al., 2005; Battilani et al., 2018; Deboever et al., 2020). Molecular oxygen can be introduced by lipoxygenase (LOX), a key enzyme involved in the oxylipin pathways, into unsaturated linolenic and linoleic acids for generating the 9- and 13-hydroperoxides, which can be further utilized as substrates by various enzymes to produce several secondary metabolites such as colneleic (CA) and colnelenic acids (CnA) (Göbel et al., 2001, 2002). These LOX-derived oxylipins display strong antimicrobial activities. The accumulation of CA and CnA is quickly increased at the pathogen-infecting sites, which confers the increased resistance of plants against pathogens (Prost et al., 2005). Moreover, application of CA to barley plants reduces disease occurrence imposed by the powdery mildew *Blumeria graminis* f. sp. hordei (Cowley and Walters, 2005). Activation of the oxylipin pathways in bean has been correlated with the induction of ISR by beneficial *Ps*eudomonas *putida* BTP1 (Ongena et al., 2004). The activities of LOX involved in the metabolic route of plant oxylipins are also enhanced in tomato plants treated with the lipopeptide-overproducing *Bacillus* strains (Mariutto et al., 2011). Therefore, the metabolism of oxylipins can be mediated by PGPR, which is involved in the regulation of plant defense responses.

## Regulation of plant induce systemic resistance by syntaxins and small RNAs

### Root hair-specific syntaxins

Root hairs play pivotal functions during nutrient and water uptake, and microbial colonization. PGPR can modify root system architecture by repressing primary root growth, and promoting root hair formation (Zamioudis et al., 2013). PGPR can also prime plant defense systems against pathogen infection (Lugtenberg and Kamilova, 2009). It has recently been indicated that root hair-specific syntaxin genes (SYPs) can mediate the PGPR-induced ISR signaling pathways in plants (Rodriguez-Furlán et al., 2016). The structure of syntaxins consists of an N-terminal auto-regulatory region, a transmembrane domain, a linker and an N-ethylmaleimide-sensitive factor attachment protein receptor (SNARE) domain (Lipka et al., 2007). The *Arabidopsis* SYP1 family contains nine proteins that are specifically localized at plasma membrane, of which can regulate plant defense responses (Uemura et al., 2004). The *SYP121* gene is involved in the regulation of exocytosis-mediated extracellular immune, which confers the enhanced plant defense against the powdery mildew *Blumeria graminis* f. sp. *hordei*, and mediates focal secretion at the pathogen-infected sites (Kwon et al., 2008). In tobacco, the *SYP132* gene regulates plant resistance against bacterial pathogens by controlling the secretion of PR1. Plant resistance to pathogens is compromised in the *syp123* mutants, which indicates that distinct plasma membrane-localized syntaxins are engaged by plants to confront pathogen infection (Kalde et al., 2007).

Cell wall in root hairs is one of the most important interacting sites with beneficial microbes. During the root hair-microbe interactions, the expression of genes encoding cell wall modifying-enzymes (e.g., pectin methyl esterase) is considerably increased for lowering cell wall rigidity (Bellincampi et al., 2014). The *SYP132*-deficient plants often display low methyl esterification in root hairs and the altered cell wall rigidity, which leads to the inhibition of plant-rhizobacteria interactions. Brisson et al. (1994) have shown that pathogenic attacks cause rapid insolubility of proline-rich proteins (PRPs) and thus strengthen the cell wall. The changes of PRP3 localization affect the activation of ISR responses in the *syp123* plants. The *prp3* mutants exhibit the increased susceptibility to the bacterial pathogen *P. syringae*. Since the expression of *PRP3* is specifically expressed in root hairs, the increased susceptibility of the *prp3* mutants confirms the idea that the proper localization of PRP3 at root cell wall is important for the PGPR-induced ISR in plants (Larson et al., 2014). Considering that plant defense actions are necessarily regulated by receptor molecules closely related to the plasma membrane, the *SYP123* gene plays other roles in the mediation of PGPR-induced ISR in plants. Abnormal localization of these receptors in the *syp123* mutants may lead to the impaired activation of ISR in plants. The induced resistance in the roots is manifested in the whole plants as the alleviation of disease severity upon the subsequent pathogen attacks. The PGPR-induced ISR partially overlaps with those of the pathogen-induced SAR in plants. Consequently, the *syp123* mutants lack the ability to mount the SAR against the bacterial pathogen *P. syringae*. Many studies have indicated that plant ISR responses are associated with the activation of defense-related genes such as *PR1*, *PDF1*.2, and *MYC2* (Ryu et al., 2003, 2004). Beneficial *Pseudomonas* species greatly increase the transcription of ISR priming marker genes as compared to the control plants, while a lower expression of these genes is observed in the *syp123* mutants (Rodriguez-Furlán et al., 2016). These results strongly indicate that root hair-specific syntaxins are essential for regulating the PGPR-induced ISR responses in plants.

### MicroRNAs

In plants, microRNAs (miRNAs) are one of the most important non-coding RNA molecules that can mediate the expression of target mRNAs by translational repression or cleavage (Baulcombe, 2004; Ha and Kim, 2014; Song et al., 2019). MiRNAs can be converted into primary miRNA, and then processed to pre-miRNAs, which contain stem-loop hairpin structures. The resulting pre-miRNAs are cleaved for producing the duplexes of miRNA/miRNA. The mature miRNAs can be loaded into an RNA-induced silencing complex (RISC), in which they bind to target mRNAs for the control of their transcription (Martinez et al., 2002; Nakanishi, 2016; Michlewski and Cáceres, 2019). In plants, miRNAs have been demonstrated to regulate various processes, such as plant growth, disease resistance, stress adaptation, and cellular signal transduction (Zhang et al., 2021; Begum, 2022). Plant-derived miRNAs also function as molecular linkers that mediate plant growth and auxin signaling pathways under adverse conditions (Sunkar et al., 2007; Padmanabhan et al., 2009; Zhang, 2015; Begum, 2022).

Recently, miRNAs have been considered as key biomarkers of plants in response to biotic factors. The expression levels of miR393 and miR167 are down-regulated in the *A. tumefaciens* C58-induced plant tumors (Dunoyer et al., 2006). Navarro et al. (2006) have reported that bacterial PAMP flg22 significantly induces the expression of miR393 in *Arabidopsis*, which is involved in the regulation of PTI responses by silencing several auxin receptors such as *TIR1*, *AFB2* and *AFB3*, and thereby weakens the auxin-mediated pathways (Navarro et al., 2006). Moreover, a non-pathogenic strain *Pst* (hrcC) enhances the expression of miR160 and miR167, which target several auxin-response factor (ARF) genes (Fahlgren et al., 2007). In addition, *Pst* hrcC infection induces the expression of miR825, which may silence several members of zinc finger homeobox gene family, remorin and frataxin-related genes. During fungal infection, the miRNA-mediated gene silencing can be employed by plants to defend against pathogenic attacks. This posttranscriptional gene silencing is essential for regulating plant defense responses against fungal pathogens (Katiyar-Agarwal and Jin, 2010). In *Arabidopsis*, several RNA silencing mutants such as *rdr2*, *sgs2*, and *sgs3* display the increased susceptibility to *Verticillium* wilt (Ellendorff et al., 2009). During viral infection, the expression of miR158 and miR1885 is largely increased in *Brassica rapa* upon exposure to the Turnip mosaic virus (TuMV) infection. Plant miR1885 has been predicted to target a TIR-NBS-LRR gene, which positively regulates plant disease resistance (He et al., 2008).

It has been indicated that miRNAs are involved in the mediation of the process of plant-microbe interactions. The expression of miR172c is positively correlated with the efficiency of rhizobia infection and nodulation formation, indicating that miR172c acts as an important regulator for plant-rhizobium symbiosis (Nova-Franco et al., 2015; Wang et al., 2019). During the symbiosis process, miR2111 can translocate from shoots to roots, thereby regulating root symbiosis suppressors to control nodule symbiosis (Zhang et al., 2021). In *Medicago truncatula*, miR396 and miR171 can regulate plant-arbuscular mycorrhizal (AM) fungi symbiosis by silencing of the growth regulating factor and nodulation signaling pathway 2, respectively (De Luis et al., 2012; Bazin et al., 2013). Rhizo-colonization of *Bacillus* strains can benefit plants through diverse means, such as secretion of auxin and antibiotic substances, the increased bioavailability of nutrients and stimulation of ISR (Abriouel et al., 2011; Shao et al., 2015; Zhou et al., 2019). Several miRNAs have recently been reported to control the process of PGPR-induced ISR in plants (Figure 2D). In *Arabidopsis*, the inhibited transcription of miR846 by *B. velezensis* FZB42 leads to the increased expression of target jacalin lectin genes and the activation of ISR by the JA signaling pathway (Xie et al., 2018). The expression of miR825/miR825* is also remarkably suppressed by *B. cereus* AR156, which leads to the stimulation of ISR in plants (Niu et al., 2016b). In *Arabidopsis*, miR472 also takes part in mediating the *B. cereus* AR156-induced ISR of plants against *Pst* by the nucleotide-binding site and leucine-rich repeat type (NBS-LRR)-mediated basal immunity (Jiang et al., 2020). However, it remains unclear how PGPR can regulate the expression of miRNAs for inducing disease resistance in plants. More recently, inoculation of tomato plants with *B. subtilis* SL18r enhances the resistance against *B. cinerea* by activating the expression of long non-coding RNA, MSTRG18363, for the decoy of miR1918, which silences the defense-related gene *SlATL20* encoding a putative RING-H2 finger gene (Zhou et al., 2021).

## Future perspectives

Since the discovery that PGPR can ISR in plants (Van Peer et al., 1991; Wei et al., 1991), now about 30 years ago, accumulative knowledges have been illustrated for the mechanisms of the PGPR-induced plant ISR responses. The plant defense system can be activated for resisting various pathogenic attacks, and also be suppressed for allowing the colonization of beneficial microbes. Both aspects of plant defense mediation are operative in the phenomenon of plant ISR, and their interplay need to be further investigated. A large gap is how the recognition of PGPR drives whole plants to improve growth and enhance disease resistance. Massive efforts for probing into molecular dialogs between plants and ISR-inducing microbes have been made, but several puzzles need to be unlocked in future. For instance, do plant roots distinguish the signals from pathogens and beneficial microbes and make appropriate response? How are the PGPR-derived signal molecules perceived in plant roots and transformed into specific responses that prime plant defense against foliar pathogens?

## Conclusion

Here, we have made a discussion about the mechanisms underlying plants recognize beneficial microbes. PGPR can be recognized as MAMPs by diverse plant PRRs and further trigger host defense responses. For establishing mutual benefits with the hosts, PGPR have developed strategies to weaken the activation of host defense systems. Moreover, the process of the PGPR-induced ISR in plants can be regulated by root hair-specific syntaxins and non-coding RNAs. However, it remains elusive how plants balance between microbial recognition and defense activation. Additionally, the transferring mechanisms of small RNAs from roots to shoots for provoking ISR need to be deeply explored.

## Author contributions

CZ and LZ wrote the manuscript. JH and XL provided some suggestions for the manuscript. All authors contributed to the article and approved the submitted version.
