# Corrigendum: Development of plant systemic resistance by beneficial rhizobacteria: Recognition, initiation, elicitation and regulation

**DOI:** 10.3389/fpls.2022.1118073

**Published:** 2023-01-19

**Authors:** Lin Zhu, Jiameng Huang, Xiaoming Lu, Cheng Zhou

**Affiliations:** ^1^ Key Lab of Bio-Organic Fertilizer Creation, Ministry of Agriculture and Rural Affairs, Anhui Science and Technology University, Bengbu, China; ^2^ School of Life Sciences and Technology, Tongji University, Shanghai, China; ^3^ Jiangsu Provincial Key Lab of Solid Organic Waste Utilization, Jiangsu Collaborative Innovation Center of Solid Organic Wastes, Educational Ministry Engineering Center of Resource-Saving Fertilizers, Nanjing Agricultural University, Nanjing, China

**Keywords:** induction of systemic resistance, microRNAs, beneficial rhizobacteria, syntaxins, volatile organic compounds

In the published article, there was an error in **Figure 3**. There is a missing citation for acknowledgement of the figure. The corrected **Figure 3** and its caption appear below.

**Figure 3:**
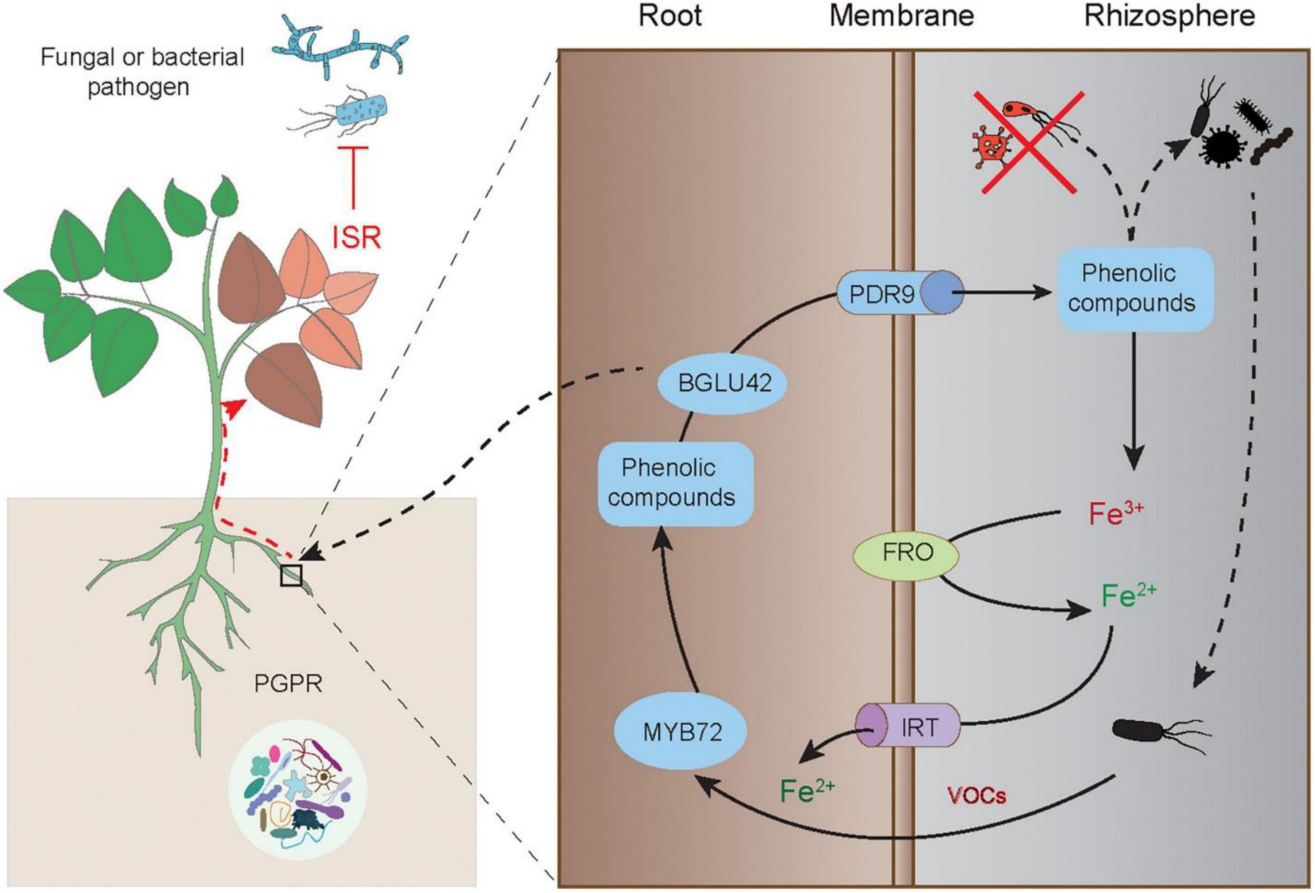
Elicitation of the MYB72-mediated ISR in plants by PGPR-released VOCs. The root-specific gene MYB72 initiates the ISR and Fe uptake in plants induced by PGPR. The ISR-eliciting PGPR can activate the expression of MYB72, which controls the biosynthesis of fluorescent phenolic compounds, and the expression of BGLU42 encoding the glucose hydrolase gene and PDR9 encoding the ABC transporter gene, thereby triggering root exudation of phenolics. The root-released phenolics further promote the mobilization of Fe^3+^ and make it available for reduction and uptake by plant roots. The phenolics can also shape specific rhizosphere microbiota. Moreover, the MYB72-dependent BGLU42 activity is essential for stimulating the ISR responses. This figure is acknowledged by Verbon et al. (2017).

This figure is acknowledged by Verbon et al. (2017).

Verbon EH, Trapet PL, Stringlis IA, Kruijs S, Bakker PAHM, Pieterse CMJ. 2017. Iron and Immunity. *Annu Rev Phytopathol*. 55:355-375. doi: 10.1146/annurev-phyto-080516-035537.

The authors apologize for this error and state that this does not change the scientific conclusions of the article in any way. The original article has been updated.

